# Pneumonic-type lung adenocarcinoma with KRAS G12V mutation and sustained response to Afatinib

**DOI:** 10.1186/s41479-024-00128-w

**Published:** 2024-04-25

**Authors:** Jie Zhao, Jiachen Xu, Tian Qiu, Jie Wang, Zhijie Wang

**Affiliations:** 1 CAMS Key Laboratory of Translational Research on Lung Cancer, State Key Laboratory of Molecular Oncology, Department of Medical Oncology, National Clinical Research Center for Cancer/Cancer Hospital, National Cancer Center, Chinese Academy of Medical Sciences and Peking Union Medical College, 17 Pan-jia-yuan South Lane, Chaoyang District, Beijing, 100021 China; 2 Department of Pathology, National Clinical Research Center for Cancer/Cancer Hospital, National Cancer Center, Chinese Academy of Medical Sciences and Peking Union Medical College, Beijing, China

**Keywords:** Pneumonic-type lung adenocarcinoma, Pneumonia, Non-small cell lung cancer, KRAS mutation, Afatinib, Next-generation sequencing (NGS)

## Abstract

**Background:**

Pneumonic-type lung adenocarcinoma (P-ADC) is a rare and challenging subtype of primary lung cancer that can be difficult to distinguish from pneumonia based on radiological images. Furthermore, no drugs are currently available that specifically target *KRAS G12V*.

**Case presentation:**

Here we report a case of P-ADC with typical and informative imaging features throughout the course of the disease, including patchy shadows, high-density lesions with aerated bronchus, diffuse ground-glass opacities, and nodular shadows from computed tomography (CT) scan. The *KRAS G12V* mutation was detected using Next-generation sequencing (NGS). An individualized Afatinib-based therapeutic schedule was prescribed and achieved sustained response after multiple lines of treatment had failed.

**Conclusion:**

Our case highlights the typical and dynamic changes in imaging features of P-ADC and provides an indicative treatment strategy for *KRAS G12V*-mutated lung adenocarcinoma.

## Introduction

Pneumonic-type lung adenocarcinoma (P-ADC) is a rare disease that typically presents with non-specific symptoms such as coughing and sputum production, occasionally accompanied by fever [1]. Laboratory examinations do not reveal any characteristic manifestations, and the imaging features often mimic the patchy shadows or consolidation of pneumonia [2]. Consequently, the diagnosis of P-ADC is frequently delayed and confused with pneumonia, especially during the Covid-19 pandemic [3, 4].

The management of P-ADC is usually similar to that of lung adenocarcinoma. For metastatic P-ADC without actionable genetic variants, chemotherapy, immunotherapy, and antiangiogenic therapy are recommended [5, 6]. Despite the recent breakthrough of Sotorasib (AMG510) targeting *KRAS G12C* mutation, there are currently no effective targeted therapies for *KRAS G12V* mutation. In this report, we present a case of typical P-ADC with *KRAS G12V* mutation that was sensitive to Afatinib, in order to provide insight for clinicians.

## Case Presentation

**Fig. 1:**
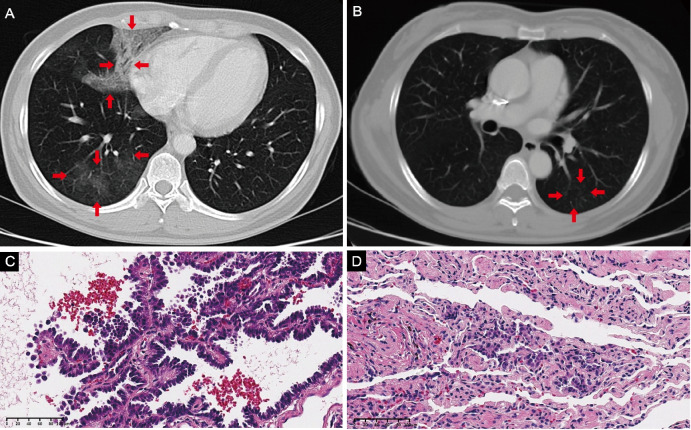
CT scan of lung-mediastinal window and pathology test. **A**, **B** Axial CT images of the chest showing pneumonia-like opacities and high-density lesions with aerated bronchus. H&E of the surgical specimens of right middle and lower lobes (**C**) and right upper lobe (**D**) confirm an adenocarcinoma

A 49-year-old woman with no smoking history presented to the clinic with patchy shadows in her lung, but she had no respiratory symptoms. On examination, laboratory tests showed a modestly elevated level of carcinoembryonic antigen (CEA) at 6.98 ng/ml, and no signs of active infection were found. A computed tomography (CT) scan showed pneumonia-like opacities and high-density lesions with aerated bronchus in the right middle and lower lobes (Fig. 1a), as well as a few pneumonia-like opacities in the left lower lobe (Fig. 1b). The percutaneous biopsy of the right middle lobe revealed lepidic adenocarcinoma. The patient then underwent a right middle and lower lobectomy, a right upper lobe wedge resection, and lymph node dissection. Pathological examination of the specimens from the right middle and lower lobes revealed a moderately to poorly differentiated adenocarcinoma with predominant lepidic growth and a small area of micropapillary pattern (Fig. 1c). The specimen from the right upper lobe showed adenocarcinoma in situ (Fig. 1d). The surgical margins were negative, and no lymph node involvement was observed (0/14). The diagnosis was primary lung adenocarcinoma (P-ADC), pT4(m)N0. Next-generation sequencing (NGS) identified *KRAS G12V* and *PTCH1 E340V* mutations, and immunohistochemical (IHC) staining indicated negative PD-L1 expression (tumor proportion score, TPS < 1%).

**Fig. 2:**
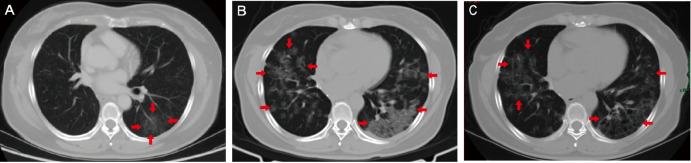
CT scan of lung-mediastinal window after four-cycles of adjuvant chemotherapy (**A**), at disease progression (**B**), and achieving lesion aera shrinkage (**C**)

**Fig. 3:**
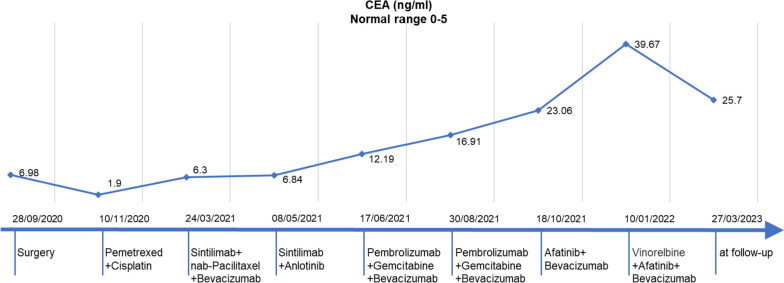
Treatment course and dynamic monitoring of CEA with time line

Following surgery, the patient received four cycles of adjuvant chemotherapy with pemetrexed plus cisplatin. However, after treatment, her CEA levels increased from 1.9 to 6.3 ng/ml, and CT scan revealed increased ground glass opacities (GGO) in the left lower lobe (Fig. 2a). Percutaneous biopsy of the left lung confirmed the presence of well-differentiated adenocarcinoma with predominant papillary morphology. NGS also revealed the presence of *KRAS G12V* and *PTCH1 E340V* mutations, and PD-L1 expression was positive (TPS 2%). Despite receiving multiple lines of treatment with chemotherapy, immunotherapy, and antiangiogenic therapy (Fig. 3), the disease continued to progress, as evidenced by markedly elevated serum CEA levels (Fig. 3) and increasing diffuse GGO, patches of high density, and nodular shadows on CT scan (Fig. 2b).

Finally, the therapeutic schedule with Afatinib, Bevacizumab plus Vinorelbine was prescribed. This new regimen resulted in shrinkage of the lesion area (improved stable disease) (Fig. 2c) and simultaneous decrease in serum CEA levels (Fig. 3). At the time of the manuscript preparation, the patient was still taking Afatinib and had achieved a progression-free survival over 14 months, with no apparent adverse effects observed.

## Discussion

The differential diagnosis between P-ADC and pneumonia is challenging, and definitive diagnosis often relies on pathological examination [7]. To date, case series of histologically confirmed P-ADC have helped summarize the imaging features. However, typical CT images of P-ADC are rarely observed in clinical practice [3]. Our case presents dynamic CT images across the entire disease stage, providing valuable insights into the imaging characteristics of P-ADC. Moreover, multiple P-ADC lesions are often histologically proven to be multi-primary lung cancers [8]. However, similar genotyping results across different lesions in this case support the conclusion of metastasis.

Targeted therapy is a well-established treatment strategy for lung adenocarcinoma with driver mutations [9]. However, drugs specifically targeting *KRAS G12V* are not yet available. Moll HP et al. [10] found that ERBB signaling was activated in human KRAS-mutated lung adenocarcinoma. Further, Afatinib, an approved pan-ERBB inhibitor, was shown to reduce KRAS-driven tumor growth in multiple mouse models, whereas erlotinib or gefitinib did not. In this case, facing failure of multiple lines of treatment and rapid disease progression, an individualized Afatinib-based therapeutic regimen achieved the best response of stable disease lasting 14 months. To our knowledge, this is the first case report of the real-world use of Afatinib for *KRAS G12V*-mutated lung adenocarcinoma, indicating a potential treatment strategy that warrants further investigation.

## Conclusions

This case presents educational CT images of P-ADC and suggests the importance of considering pan-ERBB inhibitors in clinical trials for the treatment of KRAS-mutated lung cancer.

## Data Availability

Data sharing is not applicable as no datasets were generated during the current study.

## Abbreviations

P-ADC: pneumonic-type lung adenocarcinoma
CT: computed tomography
NGS: Next-generation sequencing
CEA: carcinoembryonic antigen
IHC: immunohistochemical
